# Physiology and proteomic analysis reveals root, stem and leaf responses to potassium deficiency stress in alligator weed

**DOI:** 10.1038/s41598-019-53916-6

**Published:** 2019-11-22

**Authors:** Liqin Li, Chengcheng Lyu, Luping Huang, Qian Chen, Wei Zhuo, Xiyao Wang, Yifei Lu, Fuchun Zeng, Liming Lu

**Affiliations:** College of Agronomy, Sichuan Agricultural University, Chengdu, 611130 China

**Keywords:** Proteomics, Abiotic

## Abstract

Alligator weed is reported to have a strong ability to adapt to potassium deficiency stress. Proteomic changes in response to this stress are largely unknown in alligator weed seedlings. In this study, we performed physiological and comparative proteomics of alligator weed seedlings between normal growth (CK) and potassium deficiency (LK) stress using 2-DE techniques, including root, stem and leaf tissues. Seedling height, soluble sugar content, PGK activity and H_2_O_2_ contents were significantly altered after 15 d of LK treatment. A total of 206 differentially expressed proteins (DEPs) were identified. There were 72 DEPs in the root, 79 in the stem, and 55 in the leaves. The proteomic results were verified using western blot and qRT-PCR assays. The most represented KEGG pathway was “Carbohydrate and energy metabolism” in the three samples. The “Protein degradation” pathway only existed in the stem and root, and the “Cell cycle” pathway only existed in the root. Protein-protein interaction analysis demonstrated that the interacting proteins detected were the most common in the stem, with 18 proteins. Our study highlights protein changes in alligator weed seedling under LK stress and provides new information on the comprehensive analysis of the protein network in plant potassium nutrition.

## Introduction

Potassium (K^+^) is the most abundant inorganic cation in plant cell, takes up about 2 to10 percent of total dry weight in plant^1^ it is crucial for plant growth, development, signalling transduction, and transport processes^2^. Through K^+^ represents the fourth most abundant element in the lithosphere; however, only a low proportion (1–4%) on the surface of clay humus particles in soil is bioavailable and unavailable to plants^3^. Normally, plants accumulate large amounts of K^+^ in their vacuoles, the cytoplasmic K^+^ concentration in plant cell is ~100 mM, but the concentration near roots in the soil is very low, varying from 0.1 mM to 1.0 mM^4^. Therefore, most plants need to absorb K^+^ against the K^+^ concentration gradient from the soil. Recent research suggested K ^+^ concentration in tissue is positively correlated with plant growth, from which a critical concentration of K^+^ supporting 90% of maximum yield can be determined^5^. So potassium deficiency can directly lower various crop productivities and qualities, which may be indirectly reduced via a combination of biotic and abiotic stresses^6^.

Therefore, it is essential to make clear the molecular basis of crop responses to K^+^ deficiency and develop crops cultivars with high K^+^ -efficiency

In recent years, significant progress has been made in understanding potassium nutrition molecular mechanisms using *Arabidopsis* and rice as model plants. The Calcineurin B-like (CBL) - CBL interacting protein kinases 23 (CIPK23) complexes modulate Arabidopsis potassium transport 1 (AtAKT1) and OsAKT1 activities in the *Xenopus* oocyte^7,8^. The high-affinity K^+^ transporter 5 (HAK5) is another important high-affinity K^+^ transporter, and *AtHAK5* and *OsHAK5* were induced by low-K^+^ (LK) stress^9,10^. Previous reports indicated that the HAK5 protein can be phosphorylated and activated by CBL1 and 9-CIPK23 complexes in *Arabidopsis* roots^11^, and its expression level can be upregulated by the phosphorylation of auxin response factor 2 (ARF2) under LK stress^12^. Based on these studies, calcium-mediated CBL-CIPK complexes play a vital role in plant adaptation to K^+^ starvation.

Alligator weed (*Alternanthera philoxeroides*) is a dicotyledonous perennial herb that originated in South Americ, It is a very harmful invasive weed, some researches report it can adapt to different extreme environments because of epigenetic regulation^13,14^. Song and Su report this plant has strong capacity for K^+^ accumulation at extremely low K^+^ concentrations, this is because of its high-affinity potassium transporter^15^. When plant facing LK stress, complex morphological, physiological and biochemical processes are well controlled by K -responsive genes (proteins), thus, the identification of K^+^-responsive genes (proteins) is a fundamental step to understand the molecular mechanisms of the adaptability and plasticity of alligator weed. Some K^+^-responsive genes such as transcription factors, kinases and transporters are found in alligator weed roots by transcriptome analysis^16^.

Proteomics not only monitors protein abundance and protein interactions but also detects translational and post-translational regulations, therefore providing new insights into the changes in the plant under abiotic stress^17^. Two-dimensional polyacrylamide gel electrophoresis (2-DE) is one of the most powerful techniques.

Proteomics analysis suggests that 14-3-3, small G-proteins, NKPK and GAPDH.

May play vital roles in signal transduction pathways under LK stress in *Arabidopsis* and ramie^18,19^. Zhang *et al*. report LK stress significantly decreased the expression of most environmental-stress-related proteins; thus, K^+^ deficiency decreased the tolerance of the plant to environmental stresses in cotton^20^, Large-scale poteomics results reveal one key JA synthesis-related enzyme, allene oxide synthase (AOS), is significantly increased in K^+^-deficient wheat seedlings, further research suggest its overexpression markedly increase the tolerance of rice to LK stress, meanwhile, AOS mutant (*osaos*) exhibit more sensitivity^21^. Li *et al*. report ubiquitin pathway proteins increased significantly in roots under LK stress, and oxidative phosphorylation pathway, glycolysis/gluconeogenesis pathway, sugar metabolism and transport protein are also enhanced in stems, which could help alligator weed increase growth and survival ability under LK stress^22,23^. However, molecular mechanisms and proteomic regulatory networks remain almost unknown in alligator weed facing LK stress.

In this study, we conducted comparative proteome analysis in alligator weed roots, stem and leaves after LK stress. The objectives of this study were (i) to provides a comprehensive picture of the protein level response for LK stress in alligator weed; (ii) to find vital regulatory and functional protein to expound high LK tolerance. The results will provide a basis for further research concerning protein change and post-transcriptional response mechanisms in alligator weed under K^+^-deficiency stress.

## Results

### Effect of LK on alligator weed seedling physiology performance

**Figure 1:**
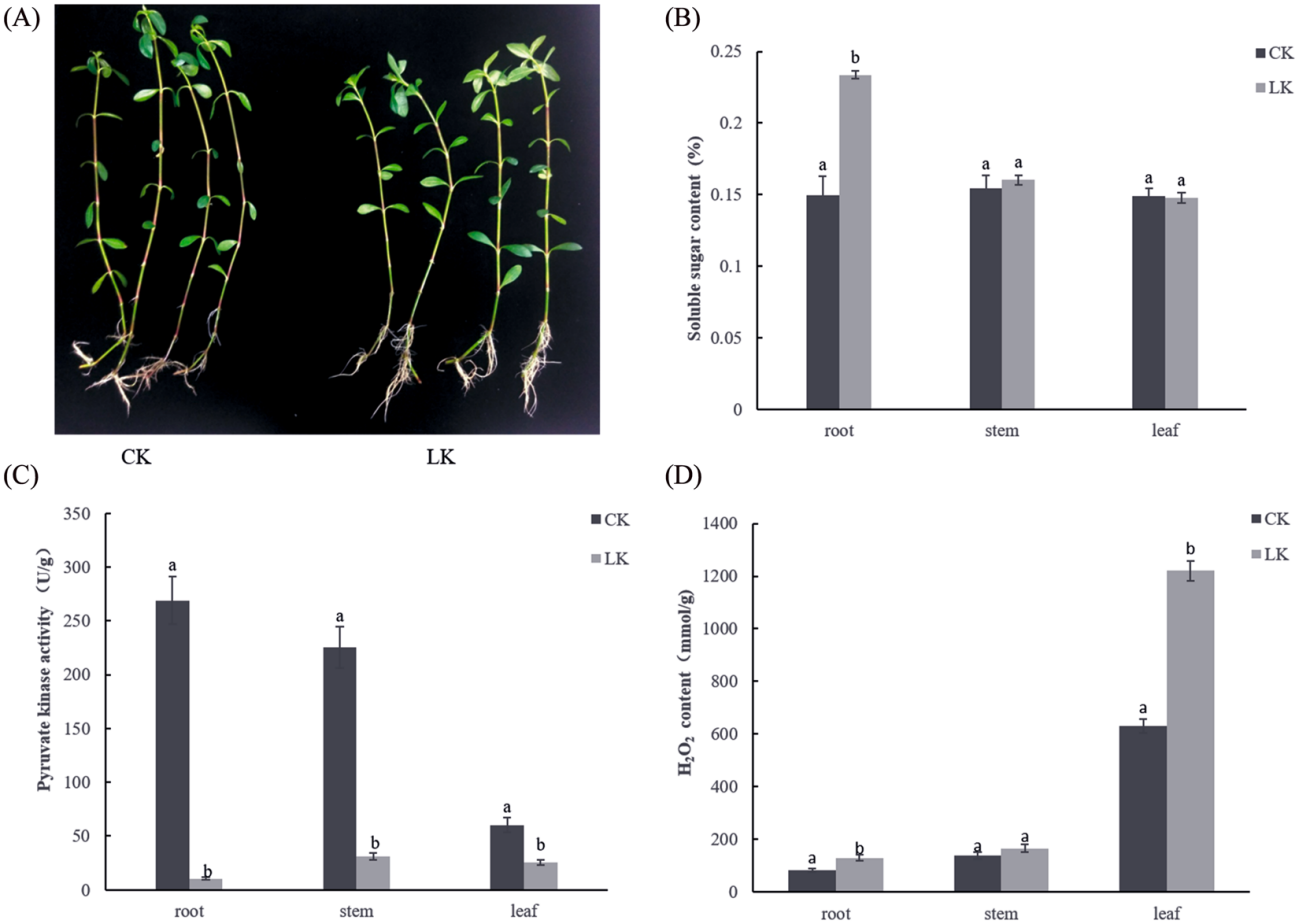
LK treatment induce growth change in Alligator weed. Phenotypes (**A**), soluble sugar content (**B)**, PK activity (**C**), H_2_O_2_ activity (**D**). Note: in all cases, the error bars show SD, and the letters indicate significant differences (Kruskal-Wallis, p < 0.05). Data are shown as the mean ± SE (n = 3), and n represents the biological replicates.

After 15 d of LK treatment, alligator weed seedlings were shorter but grew more roots than those of the CK (Fig. 1A). Root, stem and leaf samples after the CK and LK treatments were collected to conduct physiological experiments. The soluble sugar content was primarily increased in the root, but no obvious changes were detected in the stem and leaves (Fig. 1B). Pyruvate kinase (PK) activity noticeably decreased in the three samples, particularly in the root (Fig. 1C), and the H_2_O_2_ content increased in the three samples, particularly in the leaves (Fig. 1D). This suggests that after 15 d of treatment, alligator weed seedlings had a large change in their morphological and physiological characteristics.

### Proteomics response under LK stress

**Figure 2:**
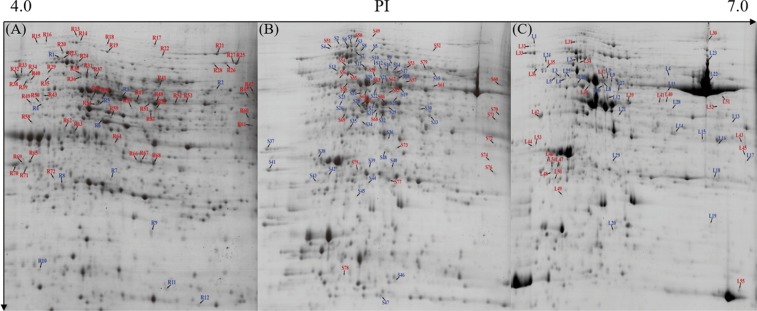
Protein change patterns in the seedlings after LK stress. The 60 DEPs with increased abundance and 12 down-regulated in the root (**A**). The 31 DEPs with increased abundance and 48 down-regulated in the stem (**B**). The 26 DEPs with increased abundance and 29 down-regulated in the leaves (**C**). Red indicates increased abundance, and blue indicates decreased abundance.

To investigate the molecular mechanism of alligator weed seedling responses to LK stress, we obtained root, stem and leaf proteome profiles using 2-DE analysis after exposure to LK stress for 15 days. All the 2-DE gels of triplicate analyses are indicated in Fig. S1. Seventy-two DEPs annotating 59 genes were found in the root, including 60 up-regulated proteins and 12 down-regulated proteins (Fig. 2A). Two spots (R13 and R14) were identified as CDC48B; two spots (R15 and R16) were heat shock proteins; two spots (R30 and R36) were ATP synthase β subunits; four spots (R32, R33, R34 and R38) were protein disulfide-isomerases; two spots (R35 and R43) were V-type proton ATPase subunits B2; two spots (R52 and R53) were ATP synthase α subunits; two spots (R62 and R63) were lignin-forming anionic peroxidases, and three spots (R66, R67 and R68) were annexins. Seventy-nine DEPs annotating 71 genes were found in the stem, including 31 up-regulated proteins and 48 down-regulation proteins (Fig. 2B). Two spots (S1 and S8) were heat shock cognate 70 kDa proteins 2; three spots (S5, S10 and S12) were RuBisCO large subunit-binding protein β subunits; five spots (S22, S24, S29, S31 and S64) were RuBisCO activase large proteins, and three spots (S20, S21 and S59) were ATP synthase β subunits. Fifty-five DEPs annotating 45 genes were found in the leaves, including 26 up-regulated proteins and 29 down-regulated proteins (Fig. 2C). Two spots (L24 and L36) were RuBisCO large subunit-binding protein α subunits; two spots (L3 and L25) were V-type proton ATPase subunits B2; two spots (L17 and L18) were carbonic anhydrases; two spots (L46 and L54) were ribose 5-phosphate isomerases A; two spots (L4 and L16) were RuBisCO large subunits; four spots (L9, L10, L26 and L39) were RuBisCO activase large isoforms; and two spots (L7 and L37) were ATP synthase β subunits. All the DEP information is listed in Tables S1–S3.

### GO analysis of DEPs identified in the 2-DE gels

**Figure 3:**
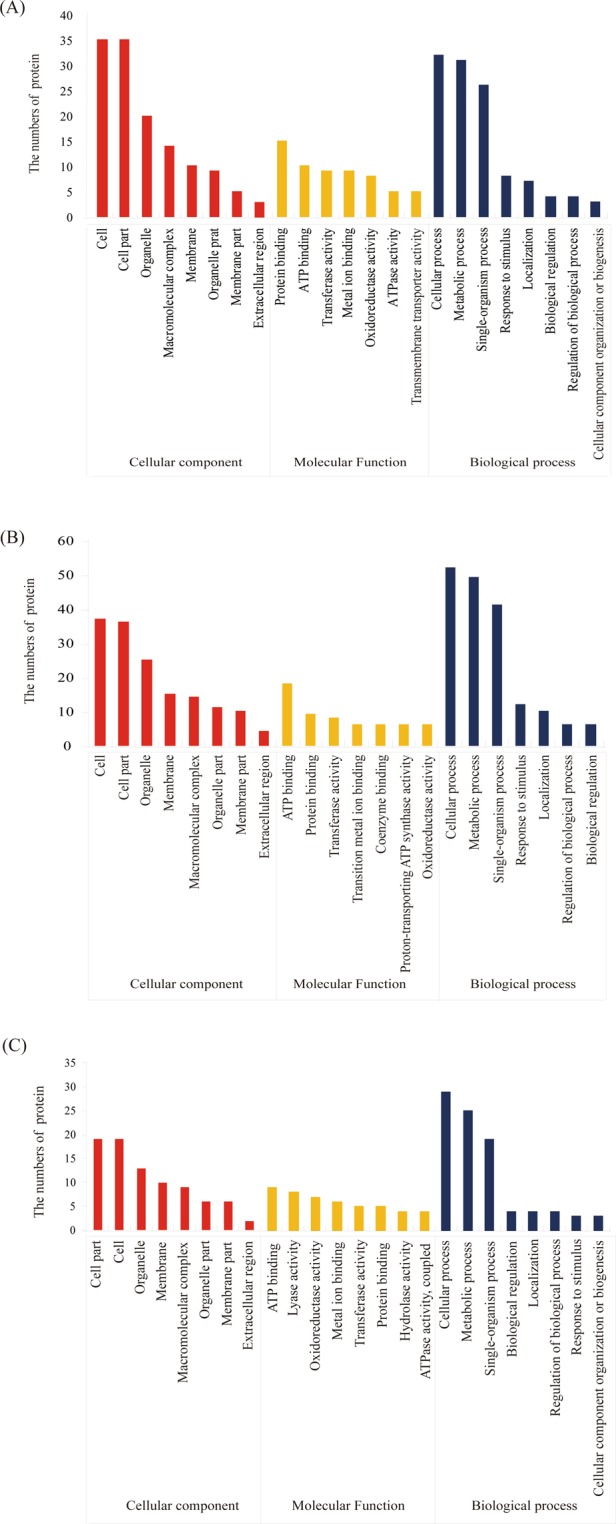
GO annotation of the DEPs in alligator weed seedlings. GO annotation in the root (**A**), GO annotation in the stem (**B**), GO annotation in the leaves (**C**).

All DEPs were annotated and classified according to biological process (BP), molecular function (MF), and cellular component (CC) using GO database. In the roots, the primary categories in CC were “Cell and cell parts”; the prominent MF categories were protein binding and “ATP binding”, and the most significant categories in BP were “Cellular process” and “Metabolic process”, followed by “Single-organism process” (Fig. 3A). In the stem, the CC ontology indicated that the major functional groups were “Cell” with 37 proteins and “Cell part” with 36 proteins. In the MF category, the major functional groups were “ATP binding” with 18 proteins and protein binding with 9 proteins. In the BP category, the major functional groups of the DEPs were involved in “Cellular process”, including 52 proteins, followed by “Metabolic process”, including 49 proteins, followed by “Single-organism process”, including 41 proteins (Fig. 3B). The CC ontology indicated that the major functional groups in the leaves were “Cell” with 19 proteins and “Cell part” with 19 proteins. In the MF category, the major two functional groups were “ATP binding” with 9 proteins and “Lyase activity” with 8 proteins. In the BP category, the major functional groups of the DEPs were involved in “Cellular process”, including 29 proteins, followed by “Metabolic process”, including 25 proteins (Fig. 3C).

### KEGG analysis of the DEPs from the 2-DE gels

**Figure 4:**
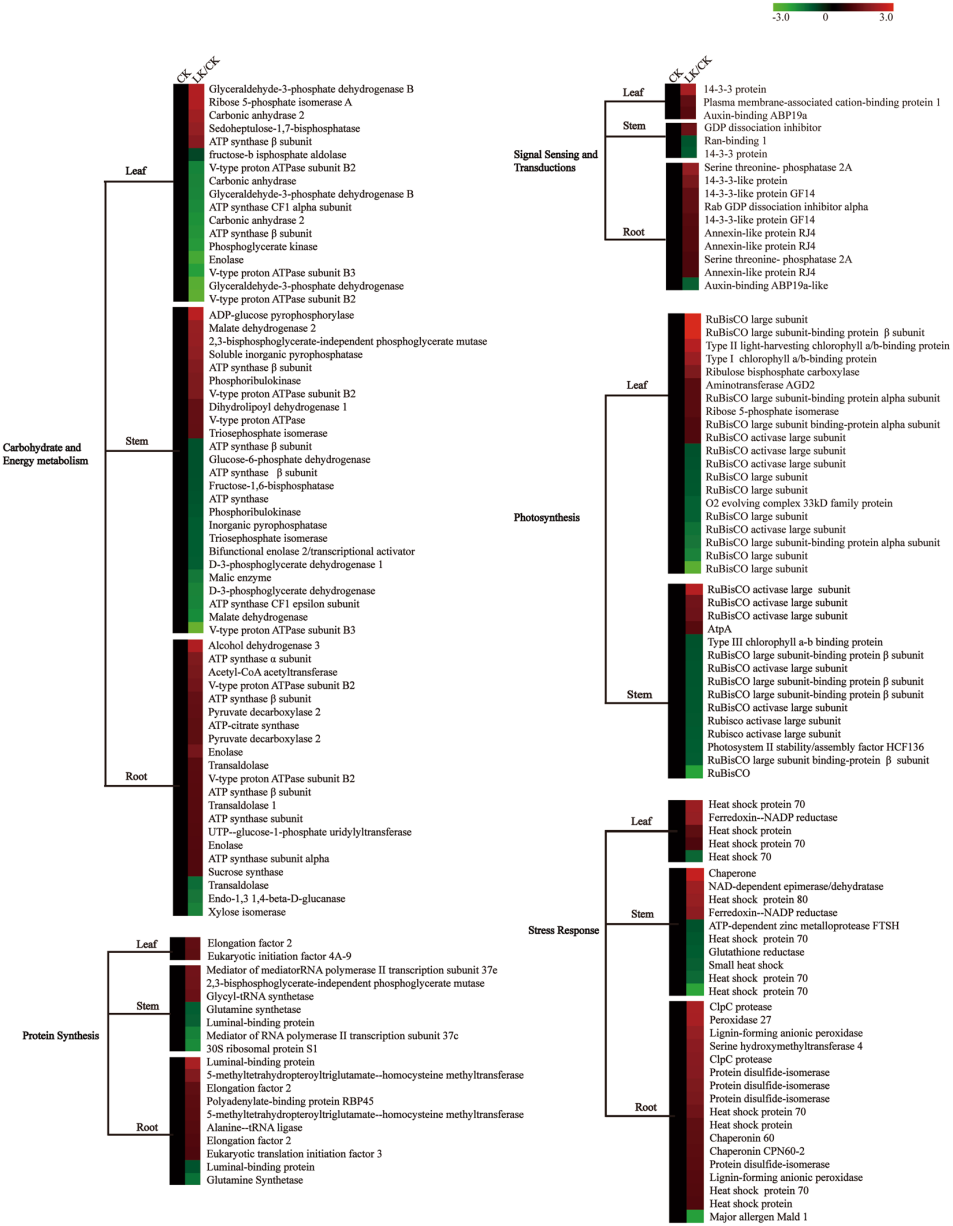
KEGG pathway analysis of DEPs in alligator weed seedlings. Red colour indicates induced proteins, and green colour indicates reduced proteins.

The KEGG pathway analyses of these DEPs were performed using the BLAST2GO 3.0 program. The most represented pathway was “Carbohydrate and energy metabolism” in the three samples, which contained 17 proteins in the leaves, 25 proteins in the stem, and 21 proteins in the root. The second was “Defense response”, which contained 5 proteins in the leaves, 10 in the stem, and 17 proteins in the root. The third was “Protein synthesis”, which contained 2 proteins in the leaves, 7 proteins in the stem, and 10 proteins in the root. The “Cytoskeleton” had the least number of proteins, which contained 1 protein in the leaves, 3 proteins in the stem, and 3 proteins in the root. “Photosynthesis” was only found in the leaves and stem, which contained 20 proteins in the leaves and 15 proteins in the stem. “Protein degradation” was only found in the stem and root, which contained 2 proteins in the stem and 3 proteins in the root. “Cell cycle” was only found in the root, including two “cell division cycle 48” proteins (Fig. 4).

### Protein-protein interaction network of DEPs

**Figure 5:**
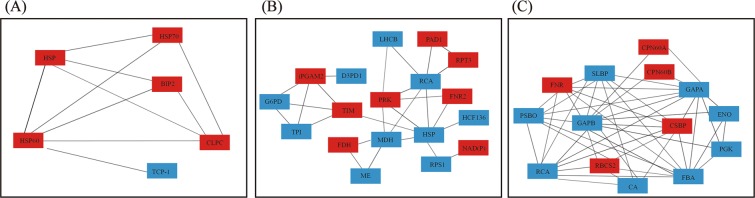
Protein-protein interaction analysis in seedlings. 6 DEPs interaction analysis in the root (**A**). 18 DEPs interaction analysis in the stem (**B**). 14 DEPs interaction analysis in the leaves. (**C**) Hsp, Heat shock protein; Hsp70, Heat shock protein 70; BIP2, Luminal-binding protein; Clpc, ClpC protease; TCP-1, T-complex 1; MDH, Malate dehydrogenase; TIM, Triosephosphate isomerase; PRK, Phosphoribulokinase; RCA, RuBisCO activase large subunit; FNR2, Ferredoxin–NADP reductase 2; HCF136, Photosystem II stability/assembly factor HCF136; PRS1, 30S ribosomal protein S1; FNR, Ferredoxin–NADP reductase 1; PSBO, O2 evolving complex 33kD family protein; RCA, RuBisCO activase large isoform; RBCS2, Ribulose bisphosphate carboxylase small chain 2; CA, Carbonic anhydrase; FBA, Fructose-bisphosphate aldolase; PGK, Phosphoglycerate kinase; CSBP, Sedoheptulose-1,7- chloroplastic; ENO, Enolase; GAPA, Glyceraldehyde-3-phosphate dehydrogenase, CPN60B, RuBisCO large subunit-binding protein subunit beta; Cpn60A, RuBisCO subunit binding-protein alpha subunit; SLBP, Stem-loop binding protein. Red blocks indicated induced protein, blue blocks indicated reduced protein.

The DEPs identified in the three samples were subjected to STRING (v10) analysis and visualized using Cytoscape 3.40 software with a high confidence to identify protein-protein interaction networks. Six proteins had interactions in the root, including 5 up-regulated proteins and 1 down–regulated protein, and HSP60 had 5 interacting proteins, including Hsp, Hsp70, BIP2, Clpc1, and TCP-1 (Fig. 5A). Eighteen proteins had interactions in the stem, with 8 up-regulated and 10 down-regulated proteins, and the HSP had 7 interacting proteins, including MDH, TIM, PRK, RCA, FNR2, HCF136, and PRS1 (Fig. 5B). Fourteen proteins had interactions in the leaves, with 6 up-regulated and 8 down-regulated proteins (Fig. 5C), including FNR, PSBO, RCA, RBCS2CA, FBA, PGK, CSBP, ENO, GAPA, CPN60B, CPN60A, and SLBP. The protein-protein interaction details were also presented in Table S4.

### Verification of transcript expression levels of DEPs

**Figure 6:**
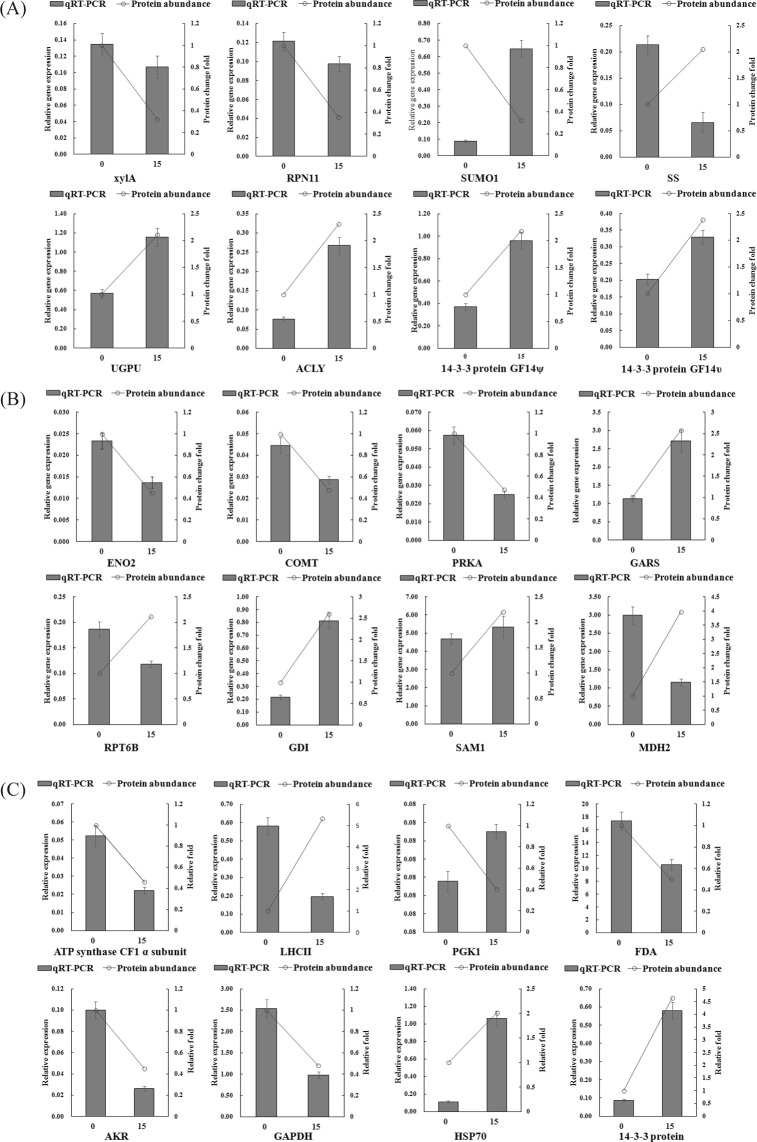
Confirmation of the proteomic results using qRT-PCR. qRT-PCR analysis in the root (**A**). qRT-PCR analysis in the stem (**B**). qRT-PCR analysis in the leaves (**C**). XylA, Xylose isomerase; PSN11, 26 S proteasome non-ATPase regulatory subunit 11; SUMO1, Small ubiquitin-related modifier 1; SS, Sucrose synthase; UGPU, UTP–glucose-1-phosphate uridylyltransferase; ACLY, ATP-citrate synthase; ENO2, Bifunctional enolase 2/transcriptional activator; COMT, Caffeic acid 3-O-methyltransferase; PRKA, Phosphoribulokinase; GARS, glycyl-tRNA synthetase; RPT6B, 26S protease regulatory subunit 6B; GDI, GDP dissociation inhibitor; SAM1, S-adenosylmethionine synthase 1; MDH2, Malate dehydrogenase 2; LHCII, Type II light-harvesting chlorophyll a/b-binding protein; PGK1, Phosphoglycerate kinase; FDA, Fructose-bisphosphate aldolase; AKR, Aldo/keto reductase; GADPH, Glyceraldehyde-3-phosphate dehydrogenase; HSP70, heat shock protein 70. Note: in all cases, the error bars show SD, Data are shown as the mean ± SE (n = 3), and n represents the biological replicates.

A total of 24 proteins were randomly selected to verify the accuracy of the 2-DE- proteomics data, and the expression of the corresponding genes was confirmed using quantitative real-time PCR(qRT-PCR). There were 8 genes detected in root (Fig. 6A), and of these, 6 gene expression patterns in different comparisons showed the same tendencies as those for protein abundance, including xylose isomerase, 26 S proteasome non-ATPase regulatory subunit 11, UTP-glucose-1-phosphate uridylyltransferase, ATP-citrate synthase, 14-3-3 protein GF14ψ, and 14-3-3 protein GF14υ; In the stem, 6 gene expression patterns in different comparisons showed the same tendencies as protein abundance (Fig. 6B), including bifunctional enolase 2/transcriptional activator, caffeic acid 3-O-methyltransferase, phosphoribulokinase, putative glycyl-tRNA synthetase, GDP dissociation inhibitor, S-adenosylmethionine synthase 1, and 26 S protease regulatory subunit 6B, and malate dehydrogenase 2 showed opposite patterns. In the leaves, the ATP synthase CF1 α subunit, fructose-bisphosphate aldolase, aldo/keto reductase, glyceraldehyde-3-phosphate dehydrogenase B, heat shock protein 70, 14-3-3 protein, and phosphoglycerate kinase showed the same patterns. Only one Type II light-harvesting chlorophyll a/b-binding showed an opposite pattern (Fig. 6C). All the primers sequences are listed in Table S5.

### Verification of protein abundance using western blot

**Figure 7:**
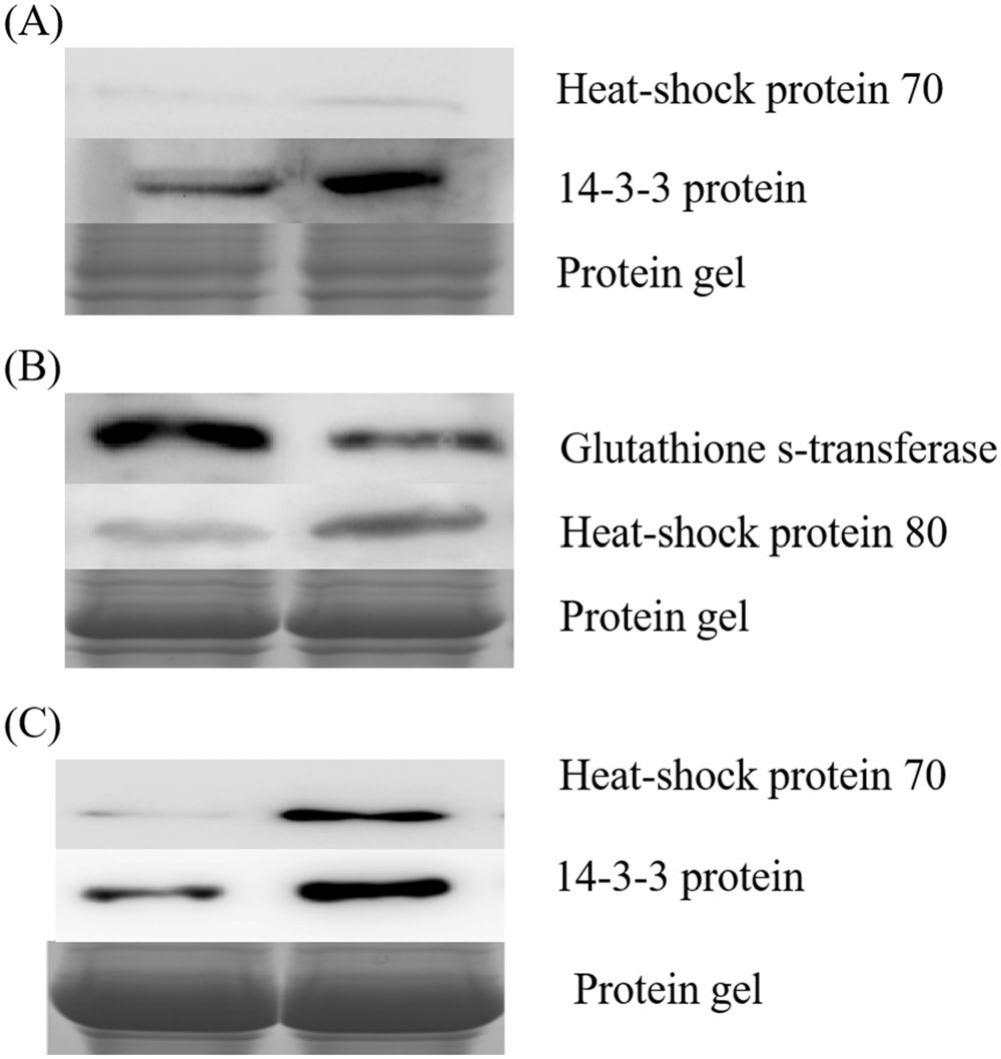
Protein abundances were examined using western blot. Western blot analysis in the root (**A**). Western blot analysis in the stem (**B**). Western blot analysis in the leaves (**C**).

Six candidate proteins were chosen to verify the protein abundance for quantification using western blot. The western blot results confirmed that the elongation factor 2 (EF2) and 14-3-3 proteins increased in abundance in the root (Fig. 7A). Glutathione reductase decreased, and HSP80 increased in abundance in the stem (Fig. 7B). The two proteins changed in a manner consistent with the proteomics results. Heat shock protein 70 (HSP70) and the 14-3-3 proteins increased in abundance in the leaves according to the western blotting results (Fig. 7C). Protein gels of root, stem and leaf for western blot was listed in Supplementary Fig. S2. Thus, the six proteins changed in a manner consistent with the proteomics results.

## Discussion

Plants require specific signal transduction to achieve vital biological functions in their life cycles. Thus, the initial LK stress signals must be transmitted by some signalling molecules and activate stress-responsive mechanisms to protect and repair damaged proteins and membranes in the plant. Plant 14-3-3 proteins are involved in diverse biological processes and considered to be vital regulators in various signal pathways. In higher plants, 14-3-3 proteins can activate vacuole two-pore K^+^ channel (TPK1) activity during potassium deficiency^24^ and also involved in salt and water stress^25,26^. Three 14-3-3 proteins in the roots and one in the leaves increased in abundance, and this result were confirmed by using qRT-PCR and western blot results in our study. 14-3-3 proteins were also found to be induced in *Arabidopsis* seedlings, tomato roots and alligator weed root under LK stress^19,23,27^. According to the current results, 14-3-3 protein plays an important and positive regulatory role in potassium signal transduction pathway, which is a conservative mechanism facing LK stress in plants.

Plant serine/threonine-protein phosphatase 2A (PP2A) is a multifunctional regulator in metabolic enzyme activities, hormone and cell cycle progression cell cycle progression. Rashotte *et al*. report PP2A plays vital roles for root auxin transport, gravity response, and lateral root development in Arabidopsis^28^. In our study, two PP2A increased in abundance in the root, and this is consistent with the results in *Arabidopsis* seedlings, wheat and alligator weed roots after LK treatment^19,22,23^.

Peroxidases (POD) are very important enzymes that are crucial for various biological processes in cells. Many peroxidase genes showed different changes during potassium deficiency in rice seedings by transcription analysis^29,30^. In our study, peroxidase 27 was increased abundance in the root after LK treatment, then we overexpressed ApPOD27 in tobacco, further research suggested the growth of leaves and roots in control plants was inhibited, but three transgenic lines plant grew better after 7 days of LK treatment. POD activity and K^+^ content were both significantly increased in three transgenic lines plant compare to control (Fig. S3), these results confirmed the close relationship between peroxidases and low-K^+^ signal sensing. Kim *et al*. reported overexpressing RCI3 (a peroxidase) showed more ROS production and high *AtHAK5* expression responsed to low potassium stress in Arabidopsis^31^. So this enzyme was a vital component of the low-potassium signal transduction pathway in plant.

Auxin Binding Protein 1 (ABP1) is considered to be a candidate auxin receptor and essential for the auxin control of cell division and cell expansion^32^, this protein is also a key regulator for root growth in *Arabidopsis*^33^, Grones *et al*. reported ABP1 demonstrate the crucial importance in activation of downstream ROP GTPases, inhibition of endocytosis and repolarization of PIN auxin transporter localization^34^. Auxin-binding protein ABP19b was decreased in alligator weed root, this protein was first found in proteomic data under LK stress, maybe reduced expression level of ABP19b had important unkown function, it needed more experiment proof in the future.

Cell division control protein 48 (CDC48) played a vital function in the cell division process, and overexpression of the fission yeast cdc25 in transgenic tobacco induced more lateral root formation^35^. More root growth were observed under 15d LK stress in alligator weed, therefore it is hypothesized that up-regulated expression of two CDC48B could play a vital function in root formation process. Unlike to transcriptome and proteomic data from *Arabidopsis* and rice under LK stress, this protein had been only found in alligator weed root, maybe the reason was related to hexaploid species with more gene copy in alligator weed.

AtRanBP1c (Ran binding protein) plays a key role in the nuclear delivery of proteins that suppress auxin action and regulate mitotic progress in the root tips^36^. The antisense expression of AtRanBP1c shows enhanced primary root growth but suppressed growth of lateral roots, so the down-regulation of Ran-binding protein 1 in the stem could induce primary root growth to facing stress in our stud. This merits further research. S-adenosyl-L-methionine synthase (SAMS) synthesizes S-adenosyl-L-methionine (SAM) from L-methionine and ATP. SAM is also a precursor for the biosynthesis of polyamines and the phytohormone ethylene, meanwhile, SAM participates in the methylation of nucleic acids and proteins. SAMS was down-regulated on transcription level during S-deficiency stress in *Arabidopsis*^37^, SAMS1 was also reduced abundance in the leave in our study, we guessed limiting the DNA methylation capacity in alligator weed would improve nutrition stress tolerance similar to *Arabidopsis*^38^.

Many DEPs involved in carbohydrate and energy metabolism were found in our study. ATP synthase catalyses ATP formation in the cell, and this protein functions in mitochondrial oxidative phosphorylation and is also a key negative regulator of plant cell death in Arabidopsis^39^. Two ATP synthase subunits alpha and three β subunits increased in abundance in the root, and two ATP synthase β subunits increased in the stem. These results suggest that alligator weed requires high energy levels to repair damage under K+-deficiency stress. Previous research indicated that V-type H^+^ transporting ATPases (VATPase) use energy to pump ions across membranes^40^. Two VATPase proteins were induced in our study, which was similar to the iTRAQ result in root^22^. Sucrose synthase (Sus) increased in abundance in the root, which was also reported in Arabidopsis, wheat and alligator weed^22,41,42^, suggesting that K+-deficiency induced changes in energy metabolism regulated by sucrose synthase. This is consistent with the increase in the soluble sugar content in the roots. Fructose-1,6-bisphosphatase (FBPase) is involved in the Calvin cycle, and FBPase decreased in abundance in the stem. This protein also down-regulated expression after high temperature stress in *P. haitanensis*^43^. The loss of the cytosolic FBPase limits photosynthetic sucrose synthesis and causes severe growth retardations in rice^44^, so it could be part of the reason why alligator weed seedlings were shorter after 15 d of LK treatment. Carbonic anhydrases activity is primarily located in the cytosol of the mesophyll cells, where it catalyses the hydration of atmospheric CO_2_ to bicarbonate for phosphoenolpyruvate carboxylase (PEPC), and two carbonic anhydrases decreased in abundance in the leaves, resulting in a reduction of photosynthesis in the leaves^45^. Thus, the growth of alligator weed seedlings was found to be inhibited. Malate dehydrogenases (MDHs) play a key role in the intracellular trafficking of reducing equivalents^46^. Peroxisomal MDH2 helps photoautotrophs manage nitrogen scarcity and high light by transmitting the redox state of the peroxisome to the chloroplast by means of the malate shuttle and H_2_O_2_-based redox signalling^47^. The high abundance of malate dehydrogenase 2 in the stem could possibly have a similar function to increase LK tolerance. This merits examination in more detail in the future.

Seventeen DEPs were involved in secondary metabolism in the roots, stems and leaves. Caffeoyl coenzyme A 3-O-methyltransferases (CCoAOMT) play important roles in lignin and flavonoids biosynthesis^48^, Two CCoAOMT were down-regulated in the stem in our study. The enzyme mRNA levels, protein levels, and enzyme activity in wheat stems were higher in lodging-resistant (H4564) and lodging-susceptible (C6001) wheat cultivars^49^, indicating that this protein has a positive role in lodging. Stem lodging-resistance was reduced after LK stress, partly because the CCoAOMT protein was reduced. Mercaptopyruvate sulfurtransferase (MST) is a vital enzyme in synthesizing H_2_S; H_2_S (an important gas molecule), activates the intracellular CDPKs, regulates the Ca^2+^ channel, and promotes the growth of the adventitious root growth of cucumbers^50^. MST was reduced in the stem in our study, and stem growth may have been less depressed by a reduction in the H_2_S content. Plant aldo-keto reductases (AKRs) participate in diverse plant metabolic reactions, including reactive aldehyde detoxification, the biosynthesis of osmolytes, secondary metabolism and membrane transport^51^. Weng *et al*. reported that one AKR could modulate the voltage-dependent shaker family potassium channels activity^52^. Since the AKRs decreased in abundance in the leaves, perhaps K^+^ channel activity was also depressed in alligator weed. This requires experimental proof.

Thirty-two DEPs were involved in stress response in the root, stem and leaves. Heat shock proteins (HSPs) mediate protein folding, stretching of newly synthesize peptide bonds, as well as correcting the wrong folding of peptide chains. In our study, one Hsp70 in the roots increased in abundance and was confirmed by western blot results. Two Hsp70s were induced in the leaves, and qRT-PCR and western blotting also confirmed this result. Overexpressing NtHSP70-1 grew better than control plant during heat stress, because this protein helps to prevent the fragmentation and degradation of nuclear DNA^53^. Hsp80 in the stem increased in abundance and was confirmed by the western blot result. The upregulation of these HSPs could prevent the denaturation of other proteins and avoid protein misfolding. Thus, this could improve the LK tolerance in alligator weed. Protein disulfide isomerases (PDIs) can catalyse disulfide bond formation in nascent secretory proteins and membrane proteins and can introduce the correct disulfide bonds into substrate proteins containing mispaired disulfides^54^. Overexpression of AtCYO1(PDIs) in the leaves induces a stay-green phenotype during darkness because of oxidative conditions favouring catabolism^55^. In our study, three PDIs increased in abundance in the root to ensure that the newly synthesized protein forms the correct disulfide bond and plays a normal function under stress.

Previous research indicates that serine hydroxymethyltransferase (SHMT1) plays a critical role in controlling the cell damage provoked by high light and salt abiotic stresses. Compared with the wild-type plants, SHMT1 mutants were smaller, lacked green leaves and accumulated a large amount of H_2_O_2_ in *Arabidopsis*^56^. SHMT4 increased in abundance in the root, so this protein played important roles in the pathway to scavenge ROS and contribute to alligator weed survival when under stress.

Previous reports suggested that new protein synthesis is blocked in the translation elongation factor 2 mutant (*los1–1*), specifically in the cold^57^. These results point to a vital role of elongation factor 2(EF2) in the low temperature signal transduction process. In our study, EF2 increased in abundance in the root and leaves. This protein was also up-regulated under high temperature stress^43^, suggesting that this protein helps facilitate new protein synthesis to improve plant survival rate under abiotic stress. Eukaryotic translation initiation factor 5 A (eIF5A) promotes the formation of the first peptide bond at the initiation of protein synthesis and displays chaperone activity. Overexpressing RceIF5A in *Rosa chinensis* results in high resistance to heat, oxidative and osmotic stresses, while in *Arabidopsis*, a decrease in the expression of three eIF5A isoforms resulted in enhanced susceptibility to these stresses^58^, two eIF increased in abundance in the root and leaves, so we hypothesized that this protein had a similar function to RceIF5A and TaeIF5A1 under abiotic stresses, because over-expressing TaeIF5A1 (*Tamarix androssowii*) in poplar plants exhibited enhanced superoxide dismutase (SOD) and peroxidase (POD) activities, lower electrolyte leakage and higher chlorophyll content under salt stress^59^.

**Figure 8:**
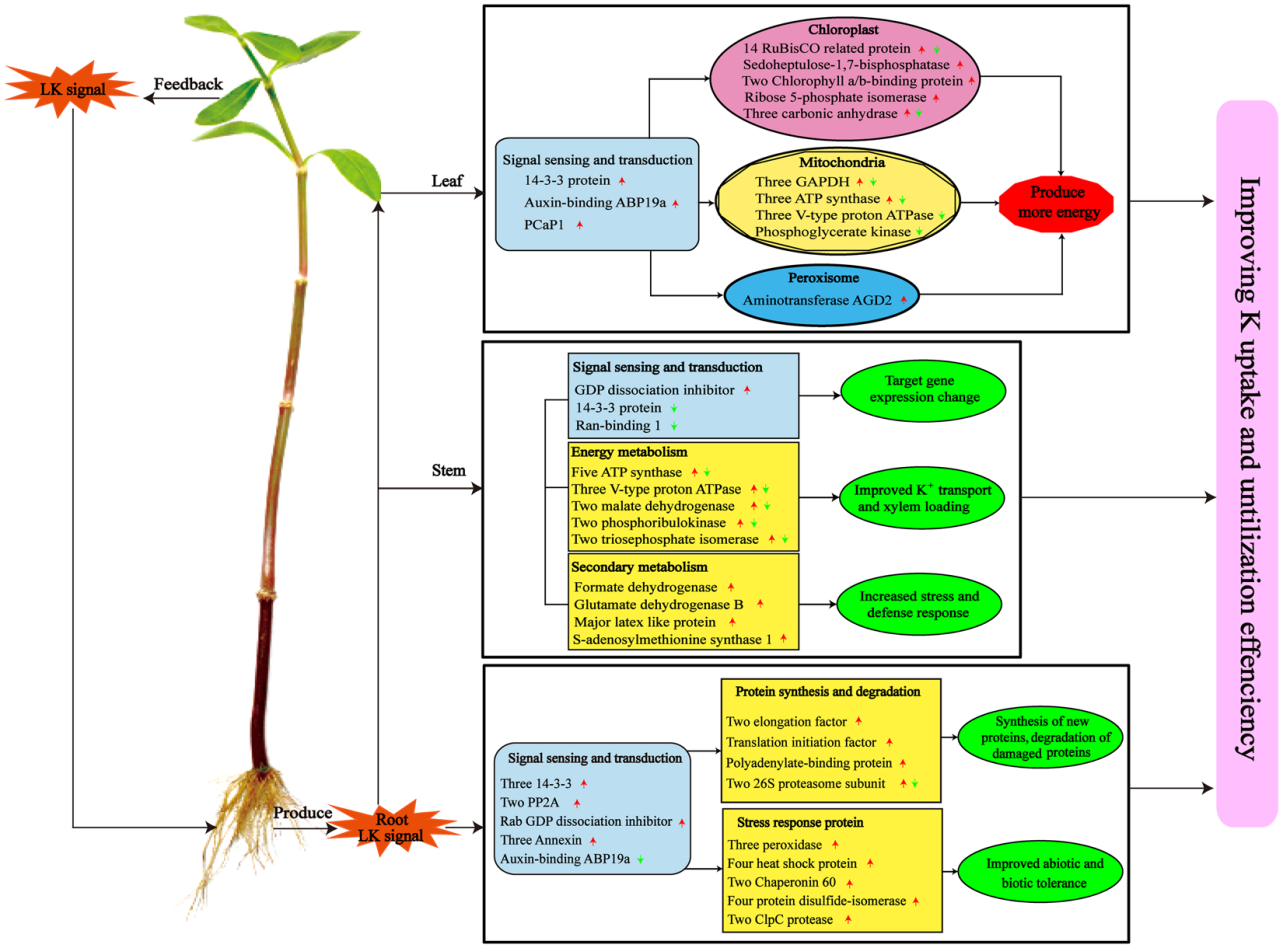
Overview of potassium deficiency -regulated protein networks in seedlings of alligator weed. PCaP1, Plasma membrane-associated cation-binding protein 1; GADPH, Glyceraldehyde-3-phosphate dehydrogenase; PP2A, Serine threonine- phosphatase 2A.

In conclusion, we conducted a comparative physiology and proteomic analysis in the roots, stems and leaves of alligator weed under LK stress. The soluble sugar content and H_2_O_2_ content were primarily increased in the root, and PGK activity were decreased in the three samples. We found there were 72 DEPs in the root, 79 in the stem, and 55 in the leaves. The abundance of some DEPs was verified using western blot assays and qRT-PCR. Bioinformatics analysis showed that most of the DEPs were involved in the “Carbohydrate and energy metabolism” and “Defense response” pathways. The “Cell cycle” pathway was only found in the root. Protein-protein interaction analysis demonstrated that 18 interacting proteins were detected in the stem, and the component HSP had 7 interacting proteins. Here we developed a protein change pathway for explaining tolerance of alligator weed under LK stress (Fig. 8).

Our experimental data provides much more new insights that could be aimed at establishing a new steady-state balance of energy metabolism and protein metabolism to response and survival under LK stress in higher plants.

## Materials and Methods

### Alligator weed sample preparation

Naturally grown alligator weed shoots were collected from a test field at the Sichuan Agricultural University (Chengdu, China), then were cultured hydroponically in a growth chamber for 10 days (d). LK treatment were conducted as previously described^15^. After 15 d of LK treatment, the root, stem and leaf samples were collected for physiological and proteomic experiments. Physiological experiments involved measuring the soluble sugar and H_2_O_2_ contents and the PK activity, and the methods of determination were conducted as previously described^60–62^.

### Plant protein extraction

Alligator weed crude proteins in root, stem and leaf were extracted using the borax/PVPP/phenol (BPP) protocol^63^. Approximately 2 g of the plant samples were ground in liquid nitrogen to cell powder and then transferred to a 5-mL centrifuge tube. Then, four volumes of lysis buffer (8 M urea, 1% Triton-100, 10 mM dithiothreitol, and 1% protease inhibitor cocktail) were added to the cell powder, followed by sonication three times on ice using a high intensity ultrasonic processor. The remaining debris was removed by centrifugation at 20,000 g at 4 °C for 10 min, and finally, the protein was precipitated with cold 20% TCA for 2 h at −20 °C. After centrifugation at 12,000 g at 4 °C for 10 min, the supernatant was discarded, the remaining precipitate was washed with cold acetone three times, the protein was redissolved in 8 M urea and the protein concentration was determined with a BCA kit according to the manufacturer’s instructions.

### Two-dimensional electrophoresis

The common 2-DE assay was performed as previously described^64^. Protein samples about 1,300 µg were diluted to a volume of 450 µL using lysis buffer which included 7 M urea, 2 M thiourea, 2% CHAPS, and 13 mM DTT. Then protein was loaded onto a 24-cm IPG strip with a linear pH gradient of 4–7 (GE Healthcare, Uppsala, Sweden) and rehydrated for 24 h at room temperature. The strips were run on an Ettan IPGphor isoelectric focusing system. The proteins were separated using 12.5% SDS polyacrylamide gels in the second dimension. Each separation was repeated 3 times to ensure the protein pattern reproducibility. The gels were staining using the GAP method, Protein spots were further analysed which Student’s t test p-values <0.05 and a relative fold change of at least 2 in their quantity.

### Protein identification via mass spectrometry and bioinformatics analysis

First, the target protein spots were manually excised and in-gel digested using bovine trypsin. Then The mass spectra of the peptides were acquired on an AB 5800 MALDI-TOF/TOF mass spectrometry (MS) instrument (AB SCIEX, Foster City, CA, USA) equipped with a neodymium laser with a laser wavelength of 349 nm. The raw MS and MS/MS spectra were combined and searched against the Viridiplantae (Green Plant) amino acid sequence database (including 11,063,808 sequences). The protein with the highest score was choosed for the bioinformatics analysis in our study. Proteins with a protein score confidence intervals above 95% (total protein score higher than 70) and more than 2.0-fold changes were considered to be confident identifications. The other evaluation criteria included at least two peptides or a coverage of 5% to accept the protein identification. In our study, samples that met two of the three criteria were considered to be trusted proteins. The sequences of the proteins identified were searched against the UniProt database (http://www.ebi.uniprot.org) to identify their functions. Hierarchical clustering of the expression profiles was performed with a self-organizing tree algorithm (SOTA) using the Cluster Software (version 3.0). Gene ontology (GO) analysis was performed by Blast2GO (http://www.blast2go.com) using the GO annotation search tool and data from the NCBI (http://www.ncbi.nlm.nih.gov) and TAIR (http://www.arabidopsis.org) databases.

### Quantitative reverse transcription PCR (qRT-PCR) and western blotting

QRT-PCR and western blot experiments were conducted to verify the proteomics results. For qRT-PCR analysis, seedlings total RNA from the control (CK) and LK were isolated using TRIzol reagent (Invitrogen, Carlsbad, USA) according to the manufacturer’s instructions. qRT-PCR was performed on a 7500 Real Time PCR System machine (Life Technologies). Actin2/8 expression was used as the internal control. The 2^−ΔΔCt^ method was used for relative quantification. The antibodies were purified using the respective oligopeptides as the affinity column tag before western blotting. Approximately 10 µg of protein was loaded per lane, and 5% non-fat milk was used to block non-specific protein binding. The nitrocellulose membrane was incubated with an anti-elongation factor 2, anti-heat shock protein 70 (HSP70), anti-HSP80, anti-14-3-3 and anti-glutathione reductase antibodies obtained from rabbit serum. Three biological replicates were performed for all western blotting results.

### Statistical analyses

For all the data from physiological and molecular experiments, at least three biological replicates were performed. We used Excel and the SPSS 14.0 statistical software package to do statistical analysis. In all cases, the error bars show SD, and the letters indicate significant differences (Kruskal-Wallis, p < 0.05).

## Supplementary information

Supplementary information
