# Supplementary material for: Physiology and proteomic analysis reveals root, stem and leaf responses to potassium deficiency stress in alligator weed
Source: Sci Rep. 2019 Nov 22;9:17366. doi: 10.1038/s41598-019-53916-6 (PMC6874644; doi:10.1038/s41598-019-53916-6)
Supplement: Supplementary file 1 — Supplementary information [file 41598_2019_53916_MOESM1_ESM.docx]

**Physiology and proteomic analysis reveals root, stem and leaf responses to potassium deficiency stress in alligator weed**

Liqin Li, Chengcheng Lyu, Luping Huang, Qian Chen, Wei Zhuo, Xiyao Wang, Yifei Lu, Fuchun Zeng & Liming Lu*

College of Agronomy, Sichuan Agricultural University, Chengdu ,611130, China.

*Corresponding authors: Dr. Liming Lu

College of Agronomy , Sichuan Agricultural University ,Huiming Road 211 number, Wenjing District, Chengdu, Sichuan 611130, China.

Tel.: +86 13880286601.

E-mail: louis_luliming@126.com

**Supporting Information File:**

Supplementary Figure S1: The original 2-DE gels for root, stem and leaf.

Supplementary Table S1: DEPs information in root was listed.

Supplementary Table S2: DEPs information in stem was listed.

Supplementary Table S3: DEPs information in leaf was listed.

Supplementary Table S4: The protein –protein interaction information was listed.

Supplementary Table S5: Primers used in qRT-PCR.

Supplementary Figure S2: Protein gels of root, stem and leaf for western blot.

Supplementary Figure S3: Overexpression of ApPo27 enhances LK tolerance in transgenic tobacco plants.

**Supplementary Figure S1: The original 2-DE gels for root, stem and leaf.**

**3 gels of CK leaf**


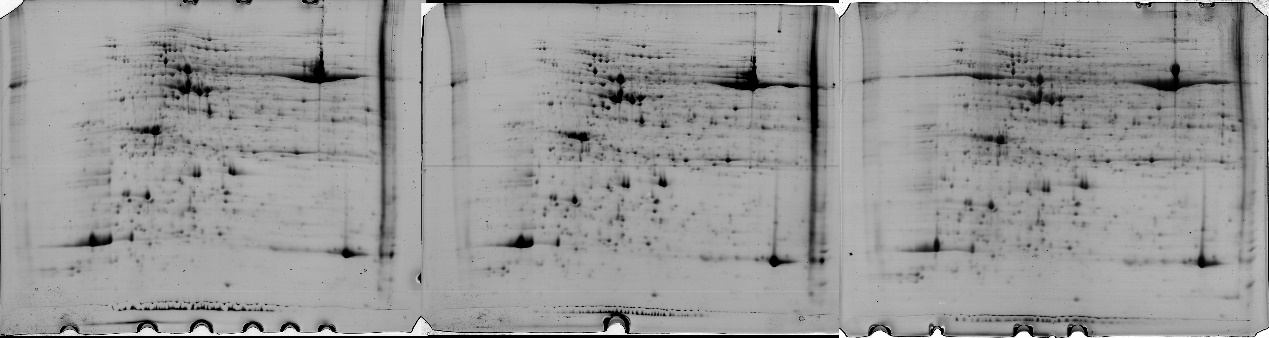


**3 gels of CK root**


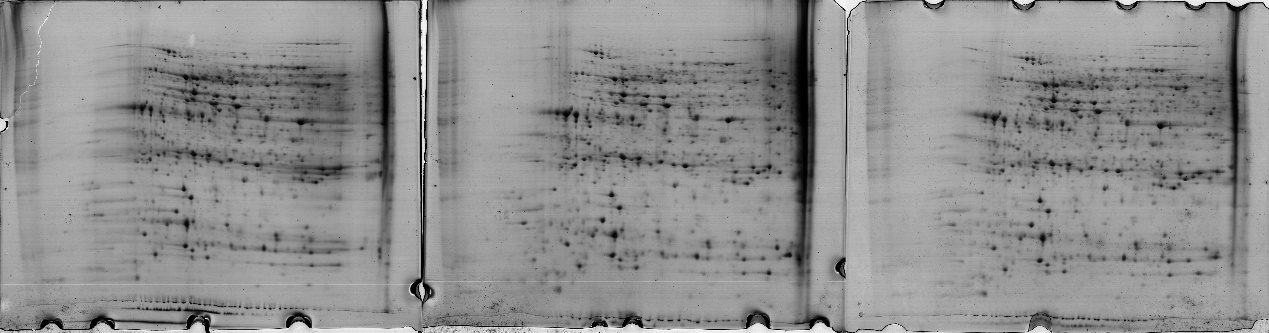


**3 gels of CK stem**

**3 gels of LK leaf**


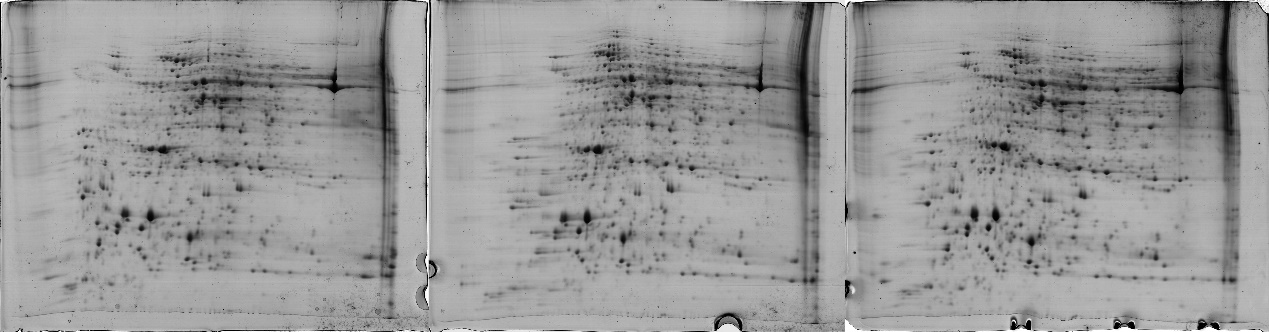


**3 gels of LK root**


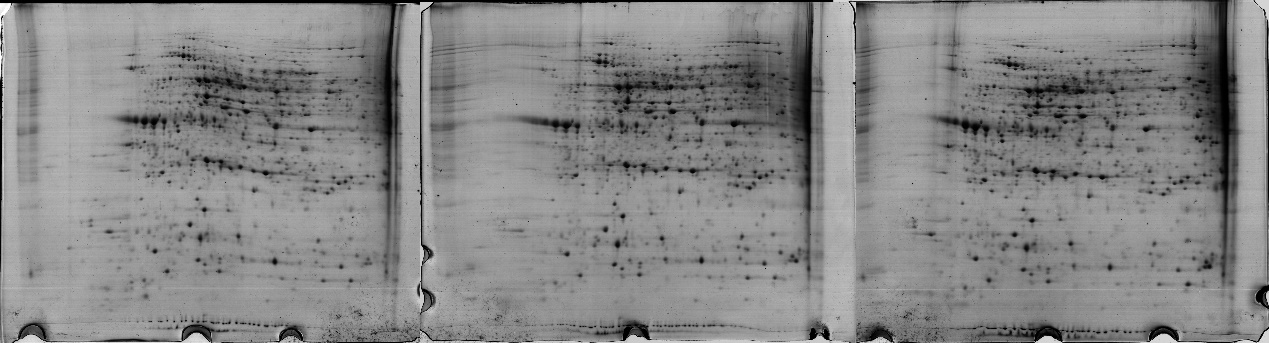


**3 gels of LK stem**


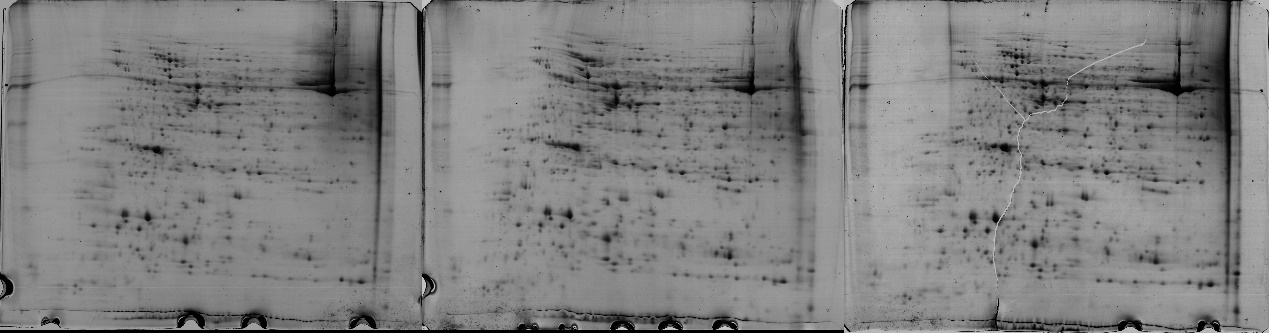


**Supplementary Table S1: DEPs information in root was listed.**

**Supplementary Table S2: DEPs information in stem was listed.**

**Supplementary Table S3: DEPs information in leaf was listed.**

**Supplementary Table S4: The protein –protein interaction information was listed.**

**Supplementary Table S5: Primers used in qRT-PCR.**

**Supplementary Figure S2: Protein gels of root, stem and leaf for western blot.**

**Supplementary Figure S3: Overexpression of ApPOD27 enhances LK tolerance in transgenic tobacco plants.**

**Plant growing on MS medium for 7 days, T1-T3 means transgenic tobacco lines.**

**
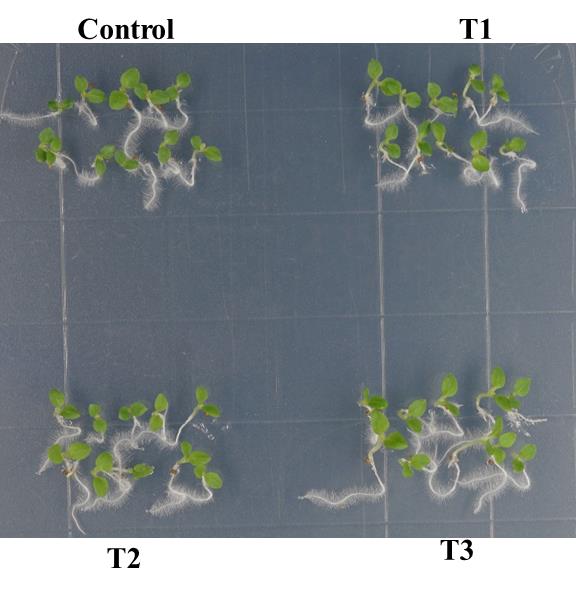
**

**Plant growing on LK medium for 7 days**

**
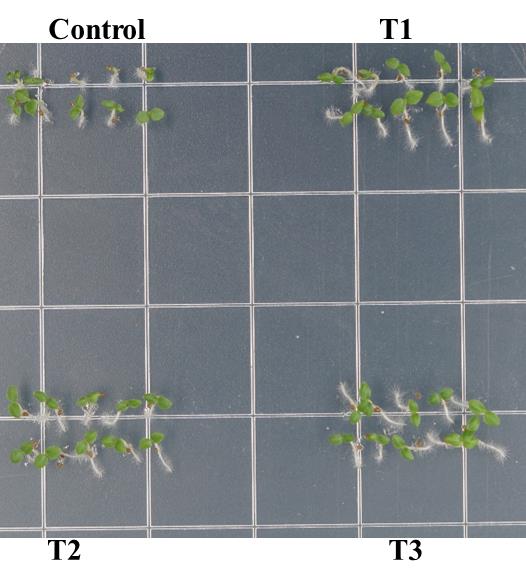
**

**Peroxidase activity**

**
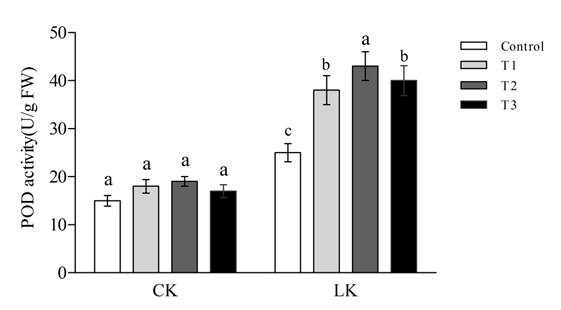
**

**Potassium content**

**
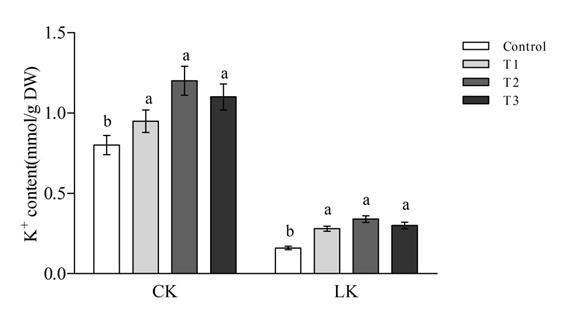
**
